# Fault Diagnosis of PMSMs Based on Image Features of Multi-Sensor Fusion

**DOI:** 10.3390/s23208592

**Published:** 2023-10-20

**Authors:** Jianping Wang, Jian Ma, Dean Meng, Xuan Zhao, Kai Zhang

**Affiliations:** School of Automobile, Chang’an University, Xi’an 710064, China; 2021022007@chd.edu.cn (J.W.); zhaoxuan@chd.edu.cn (X.Z.); zhangkai@chd.edu.cn (K.Z.)

**Keywords:** permanent magnet synchronous motors, fault diagnosis, multi-sensor information fusion, multi-signal Gramian angular difference fields, texture features

## Abstract

Permanent magnet synchronous motors (PMSMs) are extensively utilized in production and manufacturing fields due to their wide speed range, high output torque, fast speed response, small size and light weight. PMSMs are susceptible to inter-turn short circuit faults, demagnetization faults, bearing faults, and other faults arising from irregular vibrations and frequent start–brake cycles. While fault diagnosis for PMSMs offers an effective means to enhance operational efficiency, the multi-sensor information fusion is often overlooked. In industrial production processes, the collected data inevitably suffers from noise contamination, which can adversely impact diagnostic outcomes. To enhance the robustness of diagnostic methods in noisy environments and mitigate the risk of overfitting, a PMSM fault diagnosis method based on image features of multi-sensor fusion is proposed. Firstly, the vibration acceleration signals of the PMSM at different positions were acquired. Then, the newly designed multi-signal Gramian Angular Difference Fields (MGADF) method combines sensor signals from three different installation locations into a single image. Next, the multi-texture features are fused to extract the features of the image. Various machine models are compared in the fault feature learning and classification, and the results show that the proposed diagnostic method has good diagnostic accuracy and robustness, with an average diagnostic accuracy of 99.54% and a standard deviation of accuracy of 0.19. It has excellent performance even in noisy environments. The method is non-invasive and can be extended and applied to the condition monitoring and diagnosis of industrial motors.

## 1. Introduction

PMSMs are crucial electromechanical energy converters that play a significant role in diverse industrial applications due to their performance advantages, including light weight, reliable operation, low noise and high efficiency [1,2,3,4]. Typical applications in automated production lines include packaging machines, drilling machines, cutting machines, and injection moulding machines. In these applications, PMSMs drive loads with high inertia and start frequently [5]. Due to the manufacturing defects and the effects of wear, deformation and corrosion that occur during operation, the performance of the PMSM will gradually decline as component performance deteriorates, which can trigger safety hazards, and in severe cases even downtime accidents [6,7], resulting in significant economic losses [8,9,10,11]. Therefore, accurate motor fault diagnosis algorithms are crucial.

In the feature extraction stage of traditional motor fault diagnosis research, signal processing methods, including time-domain [12,13,14], frequency domain [5,15,16,17,18], and time–frequency domain [19,20,21,22,23], are commonly employed to analyze the measured signals and extract fault features associated with different states. However, the above methods often have problems of low fault diagnosis accuracy and a wide range of applications, and the related research has the limitation of extracting the detailed features of the signals in a single dimension only. However, the motor operating state signals can be converted into two-dimensional or high-dimensional space to comprehensively display the implied multi-dimensional information through multi-dimensional data fusion and high-dimensional visual knowledge methods [24].

The grayscale map coding method, while capable of partially reflecting the characteristics of vibration signals, suffers from the loss of temporal information during the coding process, leading to an absence of crucial fault characteristics. Gramian angular field (GAF) can convert the sequence signal into a 2D image, which overcomes the deficiency of missing information from gray-scale map coding, and provides a complete mapping of the signal through different features, such as colors, dots, and lines at the corresponding positions [25,26]. GAF image coding, Extreme Learning Machine (ELM), and Convolutional Neural Network (CNN) were combined to further improve the accuracy and diagnosis speed of fault classification [27]. To address the complexity of the conventional neural network structure in bearing fault diagnosis, using the construction of GAF feature maps and efficient channel attention optimization, a lightweight neural network fault diagnosis method was proposed., which achieves higher diagnostic accuracy with less parameter computation [28]. Although the above work has demonstrated diagnostic capabilities, these signal-to-image techniques are all used with a single sensor, ignoring the fusion of information from multiple sensors. It is important to recognize that single signals are more susceptible to environmental interference compared to multi-sensor signals.

In the pursuit of more accurate and stable diagnostic performance, researchers have sought to enhance their methods by combining signals from multiple sensors. Ribeiro et al. [29] employed accelerometers in two different directions to detect and diagnose six distinct types of motor faults. Their results indicate that the proposed architecture offers good accuracy in multi-sensor fault detection based on vibration time series. Gu et al. [30] proposed a correlation adaptive weighting method to integrate the collected multi-source homogeneous sensor information into multi-source heterogeneous sensor information through data layer fusion. 1D-CNN is used for feature extraction, feature layer fusion, and fault classification, and the results achieved a high fault diagnosis accuracy. Peng et al. [31] devised a motor fault diagnosis method based on a deep residual neural network (DRNN) and data fusion. Initially, they extracted time and frequency domain features from the original signal using a short-time Fourier transform (STFT) layer. Subsequently, they employed a deep residual network for feature fusion, enabling fault diagnosis via a classifier. Their method excelled in feature learning, model training, noise immunity, fault tolerance, and fault diagnosis. Yin et al. [32] proposed a fault diagnosis method combining ResNet and multi-sensor data fusion by using Fast Fourier Transform (FFT) to convert sensor data from the time-domain to the frequency domain, and by training the ResNet model for fault diagnosis and classification. However, certain challenges persist in these methods: (1) Some approaches process raw signals in the frequency and time–frequency domains, which increases fault diagnosis time and demands significant computational resources. (2) These methods transform the signal from each sensor into an image individually, leading to an increased workload during later image feature extraction and classification.

Image feature extraction methods primarily rely on artificial techniques to extract features from the underlying and middle layers by considering the various features of the image locally or globally, according to its texture, shape spatial structure and other information to carry out feature extraction. The acquired features have the advantage of strong interpretability, among which representative feature extraction methods include Tamura texture features and local binary pattern (LBP), etc. They are often applied to image scene classification. The texture is a common but difficult-to-describe feature in images, which can be regarded as an attribute displaying the pixel’s spatial distribution in the image. It is often shown as locally irregular but macroscopically regular. The traditional texture feature extraction scale is relatively single. The limited information obtained from the acquired images necessitates a description of how texture primitives are combined and arranged across multiple scales. This approach enables a more comprehensive capture of the image’s structural features and its detailed information, highlighting the unique characteristics of the image across varying scales [33].

Machine learning has become a popular technique and has been widely used in the field of motor fault detection [34]. The extracted fault features are employed in the pattern recognition stage to train machine learning models, including support vector machines, artificial neural networks, and extreme learning machines [35]. The Random Forest (RF) algorithm has a strong advantage due to its stability and resistance to overfitting. RF has a great advantage in dealing with high-dimensional data and is highly adaptable to the dataset. Secondly, RF has the advantage of fast training speed. However, the main application of RF algorithms is fault detection in induction motors and is rarely applied to PMSM fault classification.

To enhance the diagnostic method’s robustness in noisy environments and mitigate the risk of overfitting, we propose a PMSM fault diagnosis method based on the fusion of image features from multiple sensors. Firstly, the vibration acceleration signals of the PMSM at different positions under different speed and load conditions are acquired. Then, the newly designed multi-signal Gramian Angular Difference Fields (MGADF) method fuses the sensor signals from three different mounting positions into a single image, with each signal assigned to one RGB channel. Next, Tamura, HOG texture features and LBP features are fused to extract the features of the image. The effectiveness of the method is verified by experimental analysis. Multiple machine learning models are compared on fault feature learning and classification, and the results show that the diagnostic method has good performance and robustness, with excellent performance even in noisy environments. The rest of the paper is organized as follows. The theoretical background is described in Section 2. The presented method is described in Section 3. The framework of the fault diagnosis method is explained in Section 4. Section 5 explains the experiments and verifies the superiority of the proposed method by comparing it with other algorithms. Finally, the main conclusions are shown in Section 6

## 2. Theoretical Background

### 2.1. Gramian Angular Field

GAF is a method of encoding one-dimensional sequences in a way that preserves their time-dependent features to the greatest extent and maintains the signals’ dependence on time. It is a density distribution map of Gramian angular field values generated by trigonometric operations after encoding in polar coordinates. GAF overcomes the limitation of one-dimensional signals, which often represent incomplete information and fail to capture detailed features effectively. The method is not only able to maintain time dependence but also includes temporal correlation.

### 2.2. Texture Feature Extraction Method

The texture, which can be thought of as an attribute reflecting the spatial distribution characteristics of the image pixels and is frequently manifested in local irregularities and macroscopic regularity, is a common and difficult-to-describe feature in the image [20]. The texture of an image reflects the structural characteristics of the object in the image, with scale, anisotropy, rhythm and other characteristics. The traditional texture feature extraction scale is relatively single. Given the finite amount of image data available, it becomes essential to characterize the arrangement of texture primitives within an image at multiple scales. This involves combining techniques and their variations at different scales to capture more effectively both the overall structural characteristics and the finer details of the image. This approach also highlights the unique properties of images at various scales. Multi-scale texture feature extraction of images is one of the crucial techniques for image classification and recognition due to the distinct sensory characteristics of images.

### 2.3. RF

RF works by generating several classifiers that each learn and predict independently, and finally combining these results to make a prediction, which is better than the results predicted by a single classifier or model. RF consists of multiple decision trees that are independent of each other and is an integrated learning method based on these decision trees. Random Forest classification results are voted by the classification results of all the decision trees, which have a high accuracy rate [23].

## 3. The Presented Method

### 3.1. Multi-Signal Gramian Angular Fields

The GAF converts time series data into 2D images [24,25]. Firstly, the time series **X** = {*x*_1_, *x*_2_, …, *x*_4_} is scaled to the interval [−1,1] by Equation (1). ${\tilde{x}}_{i}$ indicates the value of the time series after scaling to (−1,1). The values are encoded as the angular cosine, and the timestamps are encoded as the radius *r*. The time series is retransformed to polar coordinates by using Equation (2), where *t_i_* is the timestamp, and N is a constant factor of the generated space of the regularized polar coordinate system [26].

(1) $${\tilde{x}}_{i}^{}=\frac{\left[x_{i}-\max(X)\right]+\left[x_{i}-\min(X)\right]}{\max(X)-\min(X)}$$

(2) $$\{\begin{array}{ccc} \theta =\arccos({\tilde{x}}_{i}), & -1⩽{\tilde{x}}_{i}⩽1, & {\tilde{x}}_{i}\in \tilde{X}\\ r=t_{i}/N, & t_{i}\in N & \end{array}$$

The above transformations can be used to convert the original time series into a feature map symmetric along the diagonal, which can also be used to reconstruct the time series, since the feature image contains time-related information. GAF can generate two images with different equations. Equation (3) defines the Gramian Angular Summation Field (GASF), while Equation (4) defines the Gramian Angular Difference Field (GADF). The key distinction between them is the trigonometric conversion: GASF is based on the cosine function, whereas GADF is based on the sine function.

(3) $$G_{s}=\left[\begin{array}{ccc} \cos(\theta_{1}+\theta_{1}) & \cdots & \cos(\theta_{1}+\theta_{n})\\ \vdots & \ddots & \vdots \\ \cos(\theta_{n}+\theta_{1}) & \cdots & \cos(\theta_{n}+\theta_{n}) \end{array}\right]={\tilde{X}}^{T}\tilde{X}-{\sqrt{I-{\tilde{X}}^{2}}}^{T}\sqrt{I-{\tilde{X}}^{2}}$$

(4) $$G_{D}=\left[\begin{array}{ccc} \sin(\theta_{1}-\theta_{1}) & \cdots & \sin(\theta_{1}-\theta_{n})\\ \vdots & \ddots & \vdots \\ \sin(\theta_{n}-\theta_{1}) & \cdots & \sin(\theta_{n}-\theta_{n}) \end{array}\right]={\sqrt{I-{\tilde{X}}^{2}}}^{T}\tilde{X}-{\tilde{X}}^{T}\sqrt{I-{\tilde{X}}^{2}}$$

where **I** is the unit row vector (1, 1, 1… 1); ${\tilde{X}}^{T}$ is the transpose vector of **X**. Note from the above equation that GAF is a newly constructed operation and that form corresponds to the forms of punishment of the regular inner product [35].

**Figure 1:**
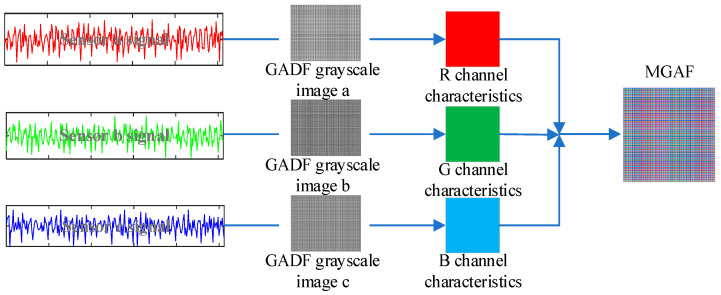
MGADF image transformation.

GADF can only transform one-dimensional time series signals, and one-dimensional signals do not provide a comprehensive enough characterization of fault features, for the signals acquired by different sensors are solved for the Gramian matrix separately and injected into the red, green and blue channels to synthesise the final MGADF image. The example of MGADF image transformation is presented in Figure 1. Due to the combination of the three sensor signals, more fault features can be represented in the MGADF image in terms of shape, texture and color features.

The innovation of the proposed method in this paper lies in the utilization of multiple sensor time-domain signals to construct the Gramian matrix, which corresponds to the features of the three channels of RGB. The image is transformed, which can further characterise the fault-specific features compared with the single–signal–source transformation method. The method further improves the differentiation between the different types of images, which creates the conditions for the improvement of the accuracy of the classification and recognition.

### 3.2. Multi-Texture Fusion for Feature Extraction

Multi-scale texture feature extraction of images has developed into one of the crucial techniques for image classification and recognition to more accurately capture the comprehensive structural features of images and their detailed information as well as to demonstrate the distinctive characteristics of images at different scales [33]. Tamura texture features include commonly used texture features such as roughness, contrast, directionality, line similarity, and so on; the LBP algorithm is a well-established image feature extraction technique known for its computational simplicity and efficiency. It is employed to describe feature descriptors related to the local texture structure of an image, reflecting the relationships between each pixel and its neighboring pixels. Notably, LBP offers significant advantages in terms of grayscale and rotational invariance; the histogram of oriented gradient (HOG) texture feature calculates the gradient feature vectors of the GAF map locally in different directions. In this paper, a feature extraction method is proposed that fuses the Tamura-HOG-LBP texture features of GAF images, which expresses the GAF spectrogram features more comprehensively from global and local texture features.

**Figure 2:**
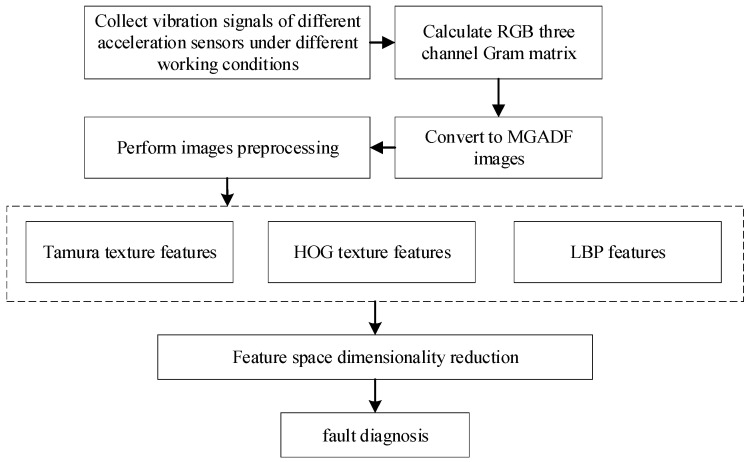
The multi-texture feature fusion extraction process.

The fusion of Tamura-HOG-LBP features improves the multi-texture feature fusion extraction process of GAF images, which is shown in Figure 2. Firstly, a PMSM fault simulation experimental platform is constructed and pre-configured with different fault types on the PMSM. Normal motors and three common typical faulty motors, including ITSF, LDF and EF motors, are designed and fabricated to carry out experiments under different operating conditions. The acceleration signals of the operation process are collected. The improved GAF image transformation is performed on the acquired signal fragments, which can comprehensively reflect the detailed features of the motors. Then, the acquired images are uniformly sized and pre-processed with a grayscale, and then the Tamura-HOG-LBP features of the images are extracted, respectively, to obtain the vector space of all the features. The dimensionality reduction is required to improve the recognition speed. Finally, pattern recognition is combined with the classifier to complete the fault diagnosis.

#### 3.2.1. Tamura Texture Feature Theory

The Tamura texture feature contains six attributes: coarseness, contrast, orientation, linearity, regularity and roughness. Coarseness indicates the granularity of the image texture pattern, the larger the granularity of the image texture pattern, the rougher the texture image, and vice versa. The coarseness calculation formula is as follows:

(5) $$T_{{}_{\mathrm{coa}}}=\frac{1}{m\times n}\sum_{h=i-1}^{i+1}\sum_{w=j-1}^{j+1}S_{\mathrm{best}}$$

(6) $$\left\{ \begin{array}{l} S_{\mathrm{best}}=\max\left\{E_{n,m}\left(i,j\right)|n\in \left[1,5\right],m=u,v\right\}\\ E_{k,u}\left(x,y\right)=|A_{k}\left(x+2^{k-1},y\right)-A_{k}\left(x-2^{k-1},y\right)|\\ E_{k,v}\left(x,y\right)=|A_{k}\left(x,y+2^{k-1}\right)-A_{k}\left(x,y-2^{k-1}\right)|\\ A_{k}(x,y)=\sum_{i=x-2^{k-1}}^{x+2^{k-1}-1}\sum_{j=y-2^{k-1}}^{y+2^{k-1}-1}\frac{g(i,j)}{2^{2k}},k=0,1,2,\ldots ,5 \end{array}\right.$$

where $A_{k}\left(x,y\right)$ is the average intensity within the active window; g(i,j) represents the gray value located there; $E_{k,u}(x,y)$ is the average gray variance of the pixels in the horizontal direction; $E_{k,\nu }(x,y)$ is the average gray variance of the pixels in the vertical direction; *m* represents the length of the image; and *n* represents the width of the image.

Contrast refers to the degree of polarization between the light and dark parts of the histogram and the dynamic range of the gray level. It could indicate the image’s clarity and the depth of the texture grooves. With increasing groove depth, the visual effect of the image becomes more contrasted and clearer. The shallower the grooves, the smaller the contrast, and the blurrier the image. The contrast is calculated as:

(7) $$T_{{}_{\mathrm{con}}}=\sigma a_{4}^{-\frac{1}{4}}$$

where $a_{4}=\frac{\mu_{4}}{\sigma^{4}}$, where $\mu_{4}$ is the quadratic moment; $\sigma^{4}$ is the variance.

The orientation degree $T_{dir}$ characterizes the pixels in the image in a certain direction. The orientation degree can be calculated by computing the gradient vector. The formula is as follows:

(8) $$T_{dir}=|d(x,y)-\mu (x,y)|$$

(9) $$\left\{ \begin{array}{l} d\left(x,y\right)=\left\{ \begin{array}{l} 0,\;|\Delta G(k,h,w)|<t\\ \theta \;(x,y),\;|\Delta G\;(k,h,w)|⩾t \end{array}\right.\\ \mu (x,y)=\frac{1}{9}\sum_{i=1}^{i+1}\sum_{j=1}^{j+1}d(i,j)\\ \left|\Delta G(x,y)|=\frac{1}{2}(|\Delta H|+|\Delta V|\right)\\ \theta =\arctan\left(\Delta V/\Delta H\right)+\pi /2 \end{array}\right.$$

where ΔH,ΔV denote the obtained horizontal and vertical gradient vector changes; d(x,y) is the direction angle; and μ(x,y) is the mean direction angle within the neighborhood;

Linearity refers to the degree of deviation of the pixel spacing distances in the calculation of the local covariance matrix. The degree of linearity is calculated as:

(10) $$T_{\mathrm{lin}}=\frac{\sum_{i=1}^{m}\sum_{j=1}^{m}P_{a}\left(i,j\right)\cos\left[\left(i-j\right)\frac{2\pi }{n}\right]}{\sum_{i=1}^{m}\sum_{j=1}^{m}P_{a}\left(i,j\right)}$$

where $P_{a}$ is the distance point of the *m* × *m* local direction covariance matrix.

Regularity is a measure of how regular an image’s texture is. The more regular the image, the closer the regularity value is to 1; otherwise, it is closer to zero. The regularity formula is:

(11) $$T_{reg}=1-r(\sigma_{coa}+\sigma_{con}+\sigma_{dir}+\sigma_{lin})$$

where *r* is the normalization factor; and $\sigma_{coa},\sigma_{con},\sigma_{dir},\sigma_{lin}$ are the standard deviations of each texture feature parameter. Roughness is used in psychology to simulate the roughness of a hand touching the surface of an object. Images of different shapes and sizes are felt differently on contact; the denser and more irregular the image, the greater the roughness, and otherwise the smaller. The roughness formula is:

(12) $$T_{rou}=T_{coa}+T_{con}$$

#### 3.2.2. HOG Texture Feature Theory

**Figure 3:**
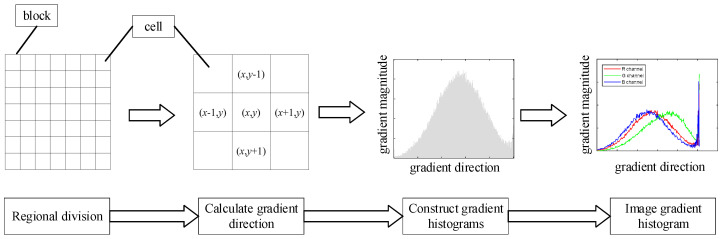
HOG feature extraction process.

The idea of the HOG algorithm is to represent the profile of an image target through the distribution of edge directions [11]. The specific strategy involves dividing the recognized image into several fixed-sized regions. In each region, gradient features are accumulated by computing the gradients of the image pixels and performing feature computation. This process yields a histogram of gradient orientation with a specified number of dimensions, as shown in the Figure 3, which is completed by the following steps:

The image is divided into two layers, and the first layer is composed of interconnected cell units. Several cells form a block, and each block can overlap. The gradient magnitude and gradient direction of a pixel point (*x*, *y*) are obtained by calculating the gradient in the coordinate direction of the point. The specific calculation formulas are shown in Equation (13):

(13) $$\left\{ \begin{array}{l} G_{x}\left(x,y\right)=H(x+1,y)-H(x-1,y)\\ G_{y}\left(x,y\right)=H(x,y+1)-H(x,y-1) \end{array}\right.$$

where $G_{x}(x,y)$·$G_{y}(x,y)$·H(x,y) denotes the gradient and pixel value of the pixel point in the *x*-axis and *y*-axis directions in the two-dimensional planar vertical coordinate system. The gradient magnitude and gradient direction at this pixel point are calculated as:

(14) $$G\left(x,y\right)=\sqrt{G_{x}{\left(x,y\right)}^{2}+G_{y}{\left(x,y\right)}^{2}}$$

(15) $$D\left(x,y\right)=\arctan\left[\frac{G_{y}\left(x,y\right)}{G_{x}\left(x,y\right)}\right]$$

The n-dimensional gradient magnitude of each cell is accumulated by dividing the gradient direction of the cell by 180 degrees equally into n blocks of directions called Bin. Multiple cells are combined into blocks for contrast normalization. HOG features are collected for all overlapping blocks in the detection window.

#### 3.2.3. LBP Feature Theory

The LBP texture analysis operator is a gray-scale invariant texture analysis method. To begin, create a 3 × 3 window that includes the value at the center of the window along with its eight neighboring values. From the upper left corner, make a clockwise size comparison. When the neighboring value is greater than or equal to the central value, record as 1, and vice versa record as 0. Through this method, a set of 8-bit binary numbers can be generated, which will be converted to decimal value through (16). The decimal value is the LBP value of this pixel point. Finally, by counting the number of occurrences of different LBP values, the image can be characterized.

(16) $$LBP\left(x_{c},y_{c}\right)=\sum_{p=0}^{7}2^{p}s\left(i_{p}-i_{c}\right)$$

where $\left(x_{c},y_{c}\right)$ is the center pixel of the 3 × 3 neighborhood; $i_{c}$ is the gray value of the center point; and $i_{p}$ is the gray value of the neighborhood pixel point.

#### 3.2.4. PCA Algorithm Dimension Reduction Processing

In the process of image recognition, if the high dimension of the original feature space is used for model training, it will increase the computational complexity greatly, and in the statistical properties of the sample cannot be estimated. Therefore, it is necessary to reduce the dimensionality of the original features. In this paper, we use the Principal Component Analysis (PCA) method to achieve feature extraction, which reduces the number of dimensions, so as to improve the speed of image recognition.

Ideally, the feature space of the sample *x* has no redundant information. The PCA algorithm can be expressed as Equation (17):

(17) $$\left\{ \begin{array}{l} y=M^{T}x\\ x=My=\sum_{i=1}^{K}y_{i}m_{i} \end{array}\right.$$

where $M=(m_{1},m_{2},\cdots ,m_{K})$ is a set of bases in the feature space, and the estimation of *x* for the first *k* terms is:

(18) $$\hat{x}=My=\sum_{i=1}^{k}y_{i}m_{i}$$

The resulting mean square error is:

(19) $$\varphi =E\left[{\left(x-\hat{x}\right)}^{T}\left(x-\hat{x}\right)\right]=\sum_{i=k+1}^{k}m_{i}^{T}E\left(x\hat{x}\right)m_{i}=\sum_{i=k+1}^{k}m_{i}^{T}Sm_{i}$$

According to the Lagrange multiplier, the expression for the extreme value of the mean square error is obtained:

(20) $$\left\{ \begin{array}{l} (S-a_{i}I)m_{i}=0,i=k+1,k+2,\cdots ,K\\ G=\sum_{i=k+1}^{K}m_{i}^{T}Sm_{i}-\sum_{i=k+1}^{K}a_{i}\left(m_{i}^{T}m_{i}-1\right) \end{array}\right.$$

where $y_{i}=m_{i}^{T}x,i=1,2,\cdots K$, ***S*** is the covariance matrix of *x*, and $m_{i}$ is the eigenvector.

The mean square error when representing x in terms of k eigenvectors is:

(21) $$\varphi =\sum_{i=k+1}^{K}a_{i}$$

From Equation (21), it can be concluded that, when the value of $a_{i}$ is smaller, the corresponding feature vector information is less impaired.

### 3.3. Random Forest Classification Algorithm with Dung Beetle Optimization

In fault classification recognition, the classifiers’ role is to determine the type of fault to which the test sample belongs based on well-labeled training data with different fault types. Linear regression and SVM are commonly used as binary classifiers and are not suitable for classifying a wide range of faults. Neural network classifiers can affect the speed of fault identification due to their slow convergence rate. Therefore, random forest is chosen as the method for classifying and identifying motor ITSC faults.

The basic structure of a random forest is a decision tree, and the main mathematical description of a decision tree is as follows: let the sample set *S* have *m* categories $c_{i}$: (*i* = 1, 2, …, *m*); $s_{i}$ is the number of samples belonging to $c_{i}$, then the sample expectation entropy is

(22) $$I(s_{1},s_{2},\cdots ,s_{m})=-\sum_{i=1}^{m}\frac{s_{i}}{s}log_{2}\frac{s_{i}}{s}$$

where *s* and $s_{i}$ denote the total number of samples and the number of samples belonging to the category $c_{i}$, respectively. For a single feature *A* of a sample, its expected entropy is:

(23) $$E(A)=\sum_{j=1}^{k}\frac{s_{1j}+s_{2j}+\cdots +s_{mj}}{s}I\left(s_{1j},s_{2j},\cdots ,s_{mj}\right)$$

where *k* denotes the total number of sample features and $s_{ij}$ denotes the *i*-th dimensional feature of the sample belonging to the category where

(24) $$I\left(s_{1j},s_{2j},\cdots ,s_{mj}\right)=-\sum_{i=1}^{m}\frac{s_{ij}}{s_{j}}log_{2}\frac{s_{ij}}{s_{j}}$$

The entropy gain of feature *A* can be obtained:

(25) $$Gain(A)=I(s_{1},s_{2},\cdots ,s_{m})-E(A)$$

The entropy gain rate ${Gain}^{\prime }(A)$ is calculated as

(26) $$Gain^{\prime }\left(A\right)=\frac{Gain\left(A\right)}{splitInfo\left(s\right)}$$

where $splitlnfo(s)=\sum_{i=1}^{m}\frac{s_{i}}{\left|s\right|}\times log_{2}\frac{s_{i}}{\left|s\right|}$.

**Figure 4:**
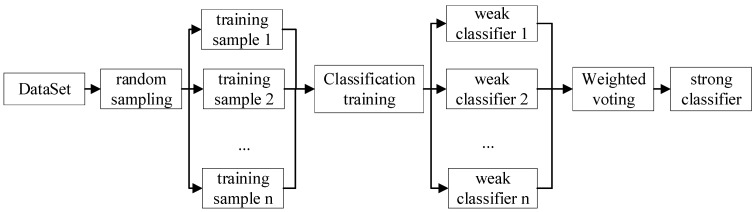
Random Forest Process.

The random forest consists of several decision tree structures, and the main process of its classification is shown in Figure 4.

Let a random forest consist of $h_{1}\left(X\right)h_{2}\left(X\right)\ldots h_{k}\left(X\right)$ decision trees, for any two features X and Y of the sample, with edge functions:

(27) $$ma\left(X,Y\right)=av_{k}\left(I(h_{k}(X)=Y)\right)-\underset{j\neq Y}{\max}av_{k}\left(I(h_{k}(X)=j)\right)$$

where *I*(.) denotes the transformation function, Y and *j* are the positive and negative categories determined by the random forest, respectively, $a\nu_{k}$ denotes the mean value, and the value of ma(X,Y) is proportional to the feature extraction effect.

RF has a great advantage in dealing with high-dimensional data and having high adaptability to the dataset. Secondly, the advantage of RF is that the training speed is quick. The importance of variables is sorted according to certain rules. The implementation is relatively simple. The idea of the RF algorithm is that a new training set is generated by randomly selecting N sample subsets from the original sample training set with put-back repetitions, and then the RF consisting of N decision trees is generated. The new classification result is obtained by judging the selection result of each class by the decision tree. The subset of elements used to decide the ideal node splits approves weaker elements’ representation in RF [36]. The low correlation between trees in RF is executed by randomization of bootstrap sampling. RF performs well in applications for rotating machinery fault diagnosis.

Dung beetle optimizer (DBO) is a population intelligent optimisation algorithm [37]. This approach is inspired by the biological behavior of dung beetles and exhibits strong optimization-seeking capabilities along with fast convergence speed. The DBO algorithm is proposed based on the rolling, dancing, foraging, stealing and breeding behaviours of dung beetles. Optimization aims to solve the number of decision trees *b* and the minimum leaf point tree *m* under the condition of satisfying the optimal solution.

When using DBO to optimise RF parameters, it is necessary to choose a suitable objective function to evaluate the advantages and disadvantages of each set of parameters. In this paper, when using RF to train each decision tree-based classifier, about 1/3 of the texture fusion feature parameters are not extracted for training, which is called Out Of Bag data. Out Of Bag data can replace the test set to estimate the generalization error of the RF model, so this paper chooses the error score rate of Out Of Bag data as the objective function of DBO for searching the optimal parameter adapted to the fault diagnosis model of the electric motor, which is calculated by the formula:

(28) $$\left\{ \begin{array}{l} \min O_{error}(m,b)=\frac{x_{w}}{x_{r}+x_{w}}\\ b\in \left[\begin{array}{c} 1,N_{1} \end{array}\right],m\in \left[\begin{array}{c} 1,M_{1} \end{array}\right] \end{array}\right.$$

where, $x_{r},x_{w}$ are the number of correctly classified and incorrectly classified samples in the out-of-bag data; *M*_1_ represents the feature attributes of the original samples; and *N*_1_ is the upper limit of the size of the decision tree-based classifier.

**Figure 5:**
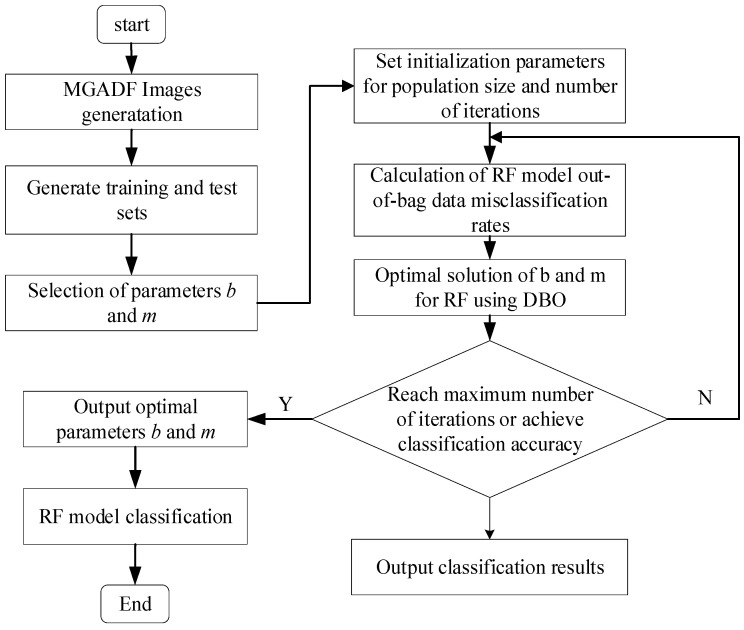
DBO-RF algorithm flow chart.

The RF and its parameter optimization process can constitute the original sample set of GAF image texture feature parameters of different states of the PMSM, proportionally divided into training and test sets, and the diagnosis of PMSM faults can be carried out based on the constructed model, which is shown in Figure 5.

## 4. Fault Diagnosis Framework

**Figure 6:**
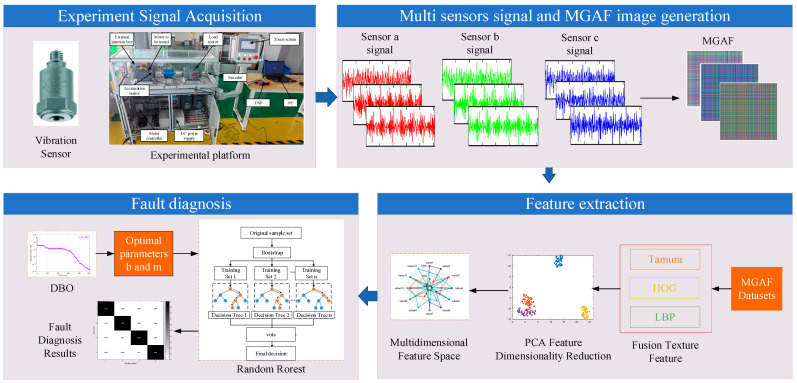
Fault diagnosis framework.

The general framework of the proposed method is shown in Figure 6 and described in detail as follows.

Step 1: Obtain Acquire time series data of PMSMs for different fault types. Measure the axial, radial and seat vibration signals of the motor in different states at different speeds and load conditions.

Step 2: Compute the Gramian matrix for each of the three signals, corresponding to the individual RGB channels, for the MGADF image transformation.

Step 3: The features of the image are solved by Tamura-HOG-LBP features.

Step 4: PCA feature space dimensionality reduction is performed on the solved features.

Step 5: The number of decision trees b and minimum leaf point tree number m under optimal solution conditions are satisfied by the DBO algorithm and fed into the RF classifier.

Step 6: Learn and generate an RF classifier to classify and identify the input features.

**Figure 7:**
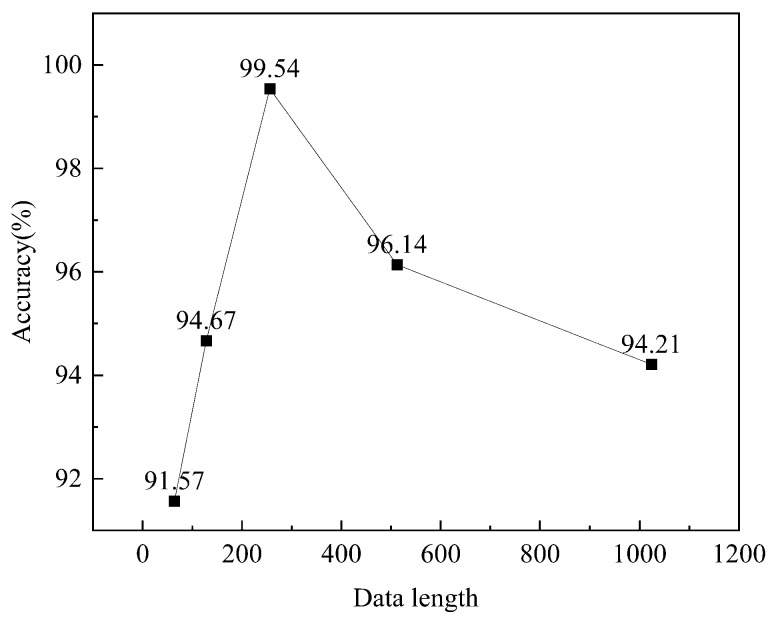
Accuracy of the different data lengths.

The length of the dataset to be coded is usually 2n, e.g., 64, 128, 256, and 512. As shown in Figure 7, the average accuracy of coding with MGADF for different data lengths is highest when the data length reaches 256. Accuracy declines after 256 bits of data are used. The data length in the following text is 256 because each pixel in the feature map created by the encoding at this point has been compressed and does not accurately represent the features of the original data.

## 5. Experimental Design

The performance of PMSMs gradually degrades as the components’ performance deteriorates, which can lead to safety hazards and, in severe cases, result in downtime accidents, causing substantial economic losses. The common types of faults in PMSM are mechanical failure, winding short circuit and demagnetization faults, etc., among which the fault characteristics of inter-turn short circuit faults, local demagnetization faults, and eccentricity faults are more similar. There has been limited research on differentiating between the three types of faults mentioned above. Therefore, this paper focuses on investigating these three fault types.

**Figure 8:**
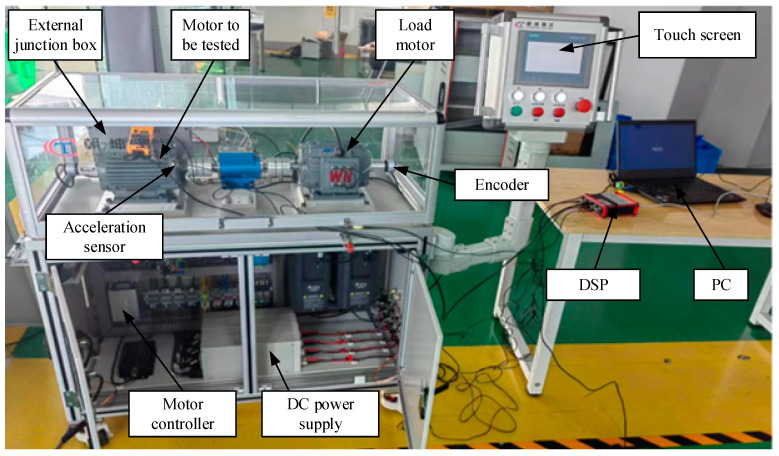
Overview of the test platform.

**Figure 9:**
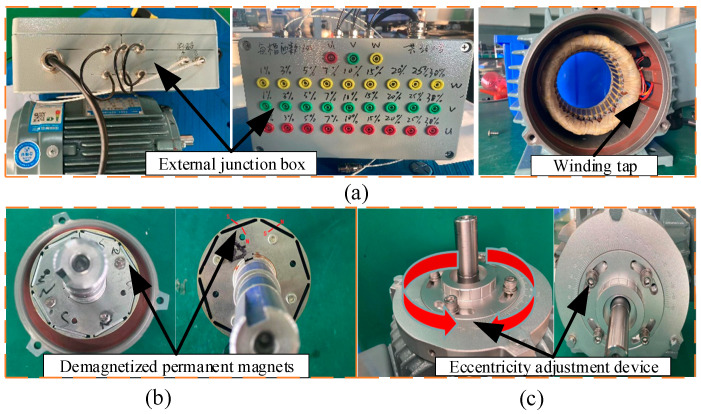
Fault implantation diagram of (**a**) ITSF, (**b**) LDF, (**c**) EF.

To verify the reasonableness of the proposed method in practical applications, a PMSM fault simulation platform is constructed, as shown in Figure 8. In the experiment, the proposed algorithm is tested on a PMSM with pre-configured faults. The main components of the experimental platform include: the PMSM to be tested, load motor, encoder, touch screen, Digital Signal Processor (DSP), personal computer (PC), direct-current power supply (DC power supply), and so on. The parameters of the PMSM to be tested are shown in Table 1, and the radial vibration data were measured, including four states (healthy state and three types of vibration signals with similar time-domain characteristics of faults): (1) Healthy Condition (HC); (2) Inter-turn Short circuit Fault (ITSF); (3) Local Demagnetization Fault (LDF); (4) Eccentricity Fault (EF). The details of the fault preset are shown in Figure 9. For the ITSF, an internal short circuit fault is simulated where the PMSM is rewound and connectors are led on 1–30% of the total number of coils in the u, v and w phase windings to an external junction box. By connecting the terminals of the junction box, ITSC faults with different numbers of turns short-circuited can be simulated on the PMSM. The experiment simulates the 20% short-circuited state of the u-phase winding; for the LDF, when a single permanent magnet is being magnetized, one of them is controlled to be magnetized up to 70% of the nominal magnetic density, and 30% of the local demagnetization is simulated; for the EF, the rotary eccentric device is designed by rotating the eccentric device of the two ends of the PMSM to achieve the adjustment of static eccentricity. The corresponding relationship between the rotation angle and the static eccentricity is a=0.8sin⁡(|θ/2|), where a is the static eccentricity, and θ is the angle of the eccentric device, which is set to 20° during the experiment. The sampling frequency of these data is 10 kHz. Each sample contains 2k points. The acceleration sensor parameters are shown in Table 2.

The number of specimens for each working condition is described in Table 3. The speeds are 1000, 1500 and 2000 r/min, and the loads are no load, half load and rated load, which are a total of nine working conditions to construct the dataset. Based on the experimental setup, there are 400 samples for each working condition. Each sample comprises signals from three different positions of the vibration sensors, and the samples in the dataset are randomly selected. Each data subset is divided into three parts for training, validation and testing, with each part being 60%, 20% and 20%, respectively.

## 6. Experimental Results

### 6.1. MGADF Images

**Figure 10:**
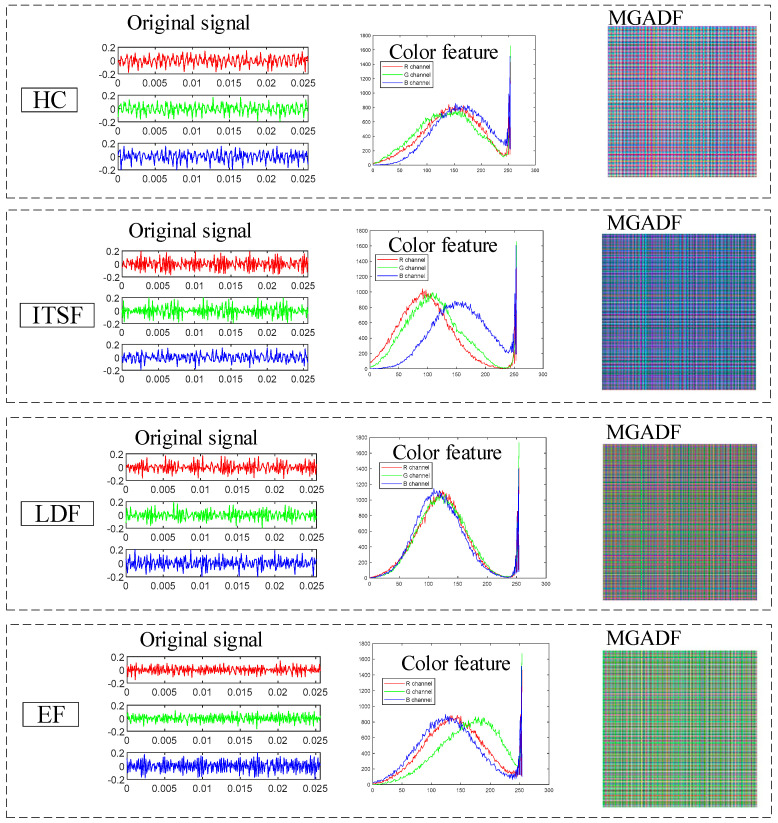
The vibration signals, color features and MGADF images.

The vibration signals, color features and MGADF images are shown in Figure 10. The vibration sensors collect red, green and blue signals from the axial, radial and seat. The time series are periodic and vary under different motor states. These signals correspond to different Gramian matrices corresponding to different RBG channels to form the MGADF. Therefore, the MGADF images are different for different fault states, showing different color characteristics. This difference is also evident when examining the RGB histogram.

**Figure 11:**
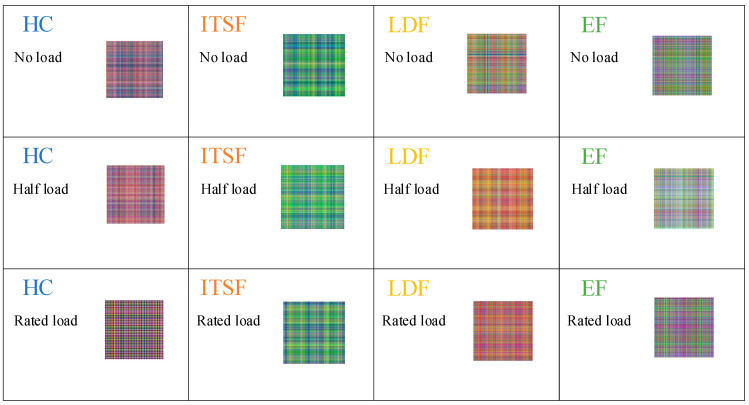
The MGADF images of HC, ITSF, LDF, and EF motors under different load conditions at the speed of 1000 r/min.

The MGADF images of the steady state dataset under different load conditions at the speed of 1000 r/min are shown in Figure 11. It is evident that there are differences between the various types, but there is a certain degree of similarity between the images of HC and LDF. The variation between the MGADF images of the same type of PMSM is not obvious as the load changes from no load to rated load.

**Figure 12:**
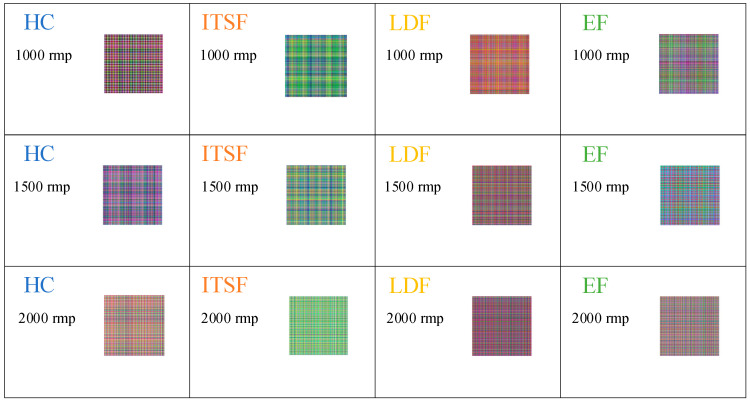
The MGADF images of HC, ITSF, LDF, and EF motors under different speed conditions with the load of rated load.

The MGADF images under different speed conditions with a load of rated load are shown in Figure 12. The images exhibit intra-class inconsistency, displaying significant variability in the MGADF images as the speed changes. Meanwhile, the HC and LDF images remain somewhat similar, increasing the inter-class ambiguity. Therefore, the above problems increase the difficulty of accurately diagnosing the fault.

### 6.2. Fusion Texture Feature Extraction

**Figure 13:**
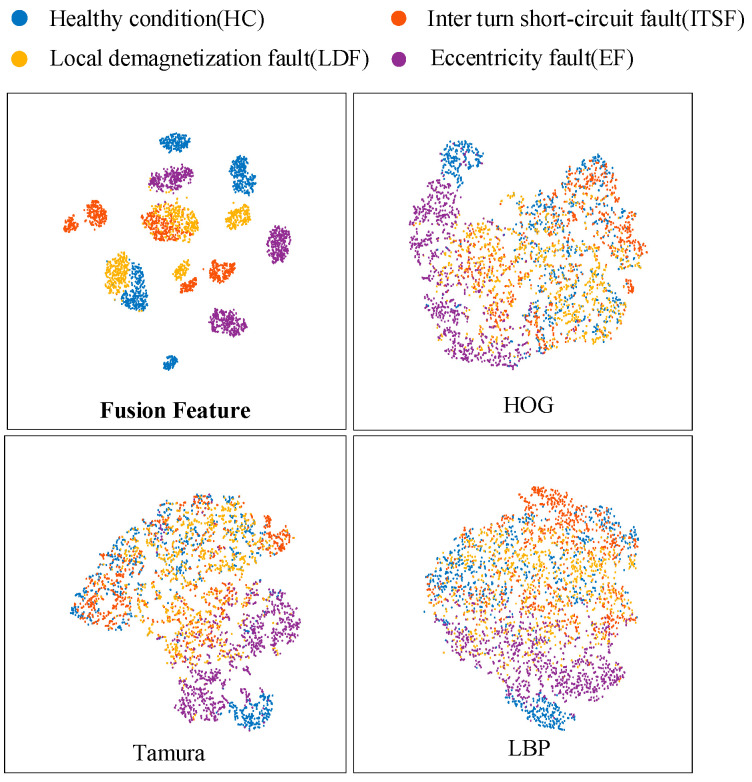
Feature visualisation for different feature extraction methods.

Based on the dataset, feature vectors are extracted by the fusion of Tamura-HOG-LBP texture features. High-dimensional data visualization is made possible by t-SNE. When we apply t-SNE to n-dimensional data, it will intelligently map n-dimensional data to 3D or even 2D data, effectively preserving the relative similarity of the original data. Because t-SNE follows nonlinearity rather than linearity, it is able to capture the intricate flow structure of high-dimensional data. In Figure 13, it can be observed that the features extracted by HOG become distinguishable to some extent and the features extracted by Tamura and LBP are less distinguishable. Of all the methods, feature fusion combines the strengths of all three texture feature extraction methods and proves to be the most effective in distinguishing the features associated with PMSM faults.

### 6.3. Analysis of Fault Diagnosis Results

#### 6.3.1. Comparison with Other Classification Methods

To verify the superiority of the deep networks in this paper, Support Vector Machines (SVM), BP Neural Networks (BPNN), Radial Basis Function Neural Networks (RBFNN), CNN, RF and DBO-RF are compared. The input data for both this method and other deep learning methods are fused texture features extracted from MGADF images, and the primary focus of the comparative experiments lies in accuracy, precision, recall, and Fl-score. In tables concerning diagnostic performance, bold text highlights the highest diagnostic results.

Table 4 presents the classification results of the different methods. It is evident that the proposed method attains the highest values for accuracy, precision, recall and F1 value, with mean values of 99.54%, 99.56%, 99.54% and 99.58%, respectively. From the table, it is evident that the diagnostic accuracy of DBO-RF is improved as compared to RF. In terms of overall performance, DBO-RF outperforms all other classifiers. It has the highest classification performance along with high algorithmic stability. Meanwhile, the method in this paper still maintains a large advantage over BP and RBF methods. In summary, the DBO-RF created in this paper has a greater advantage in intelligent diagnosis methods based on MGADF for image coding.

#### 6.3.2. Comparison with Other Signal-to-Image Methods

**Figure 14:**
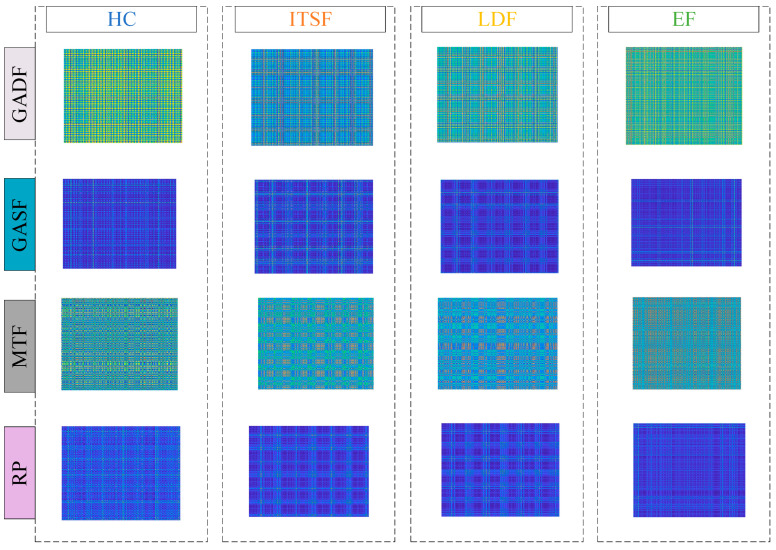
GADF, GASF, MTF and RP images with rated load and 1000 r/min.

This section compares various signal-to-image methods, including GADF, GASF, Markov Transition Fields(MTF), and Recurrence Plot(RP). In Figure 14, images are formed from an axial vibration signal with rated load and the speed of 1000 r/min. These visuals are created through point projections, which means that their colors and shapes lack interpretability. From these images, high-frequency noise can be observed.

The diagnostic results are presented in Table 5, with ‘a’ ‘b’ and ‘c’ denoting the axial, radial, and seat of the motor, respectively. Obviously, the four indicators of MGADF are the best. The four metrics are 99.54%, 99.56%, 99.54% and 99.58%. Among the other four signal-to-image methods, GADF-a has the highest average accuracy of 98.86%, which is 0.68% lower than the proposed method. The lowest average accuracy is 91.66% for RP-b, which is 7.88% lower than MGADF.

The average generation time for each image is listed in Table 6. The most efficient method is GADF, which takes 0.1152 s. This is followed by GASF and MGADF, which take 0.1153 s and 0.1221 s. All other signal image transformation methods take more time than the above methods. Hence, the method’s superiority is substantiated on the grounds of both reliability and efficiency. It can achieve reliable motor diagnosis under varying speed and load conditions.

#### 6.3.3. Effect of Signal-to-Noise Ratio

In order to avoid overfitting, which leads to high algorithm accuracy, the noise immunity of this paper’s algorithm is also investigated [38]. To simulate data collected from the same type of motor in different environments, Gaussian white noise with different signal-to-noise ratios is added to the original test dataset [39]. The signal-to-noise ratio is defined as when the signal-to-noise ratio is set to 5, 10, or 20 dB.

(29) $$\mathrm{SNR}(\mathrm{dB})=10\log_{10}\left(\frac{P_{\mathrm{signal}}}{P_{\mathrm{noise}}}\right)$$

where $P_{signal}$ denotes the power of the original signal and $P_{noise}$ denotes the power of the noise signal.

**Figure 15:**
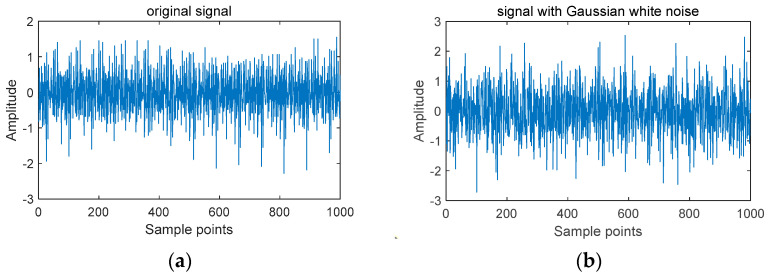
Comparison of original signal and after the adding of Gaussian white noise. (**a**) Original signal. (**b**) Signal with Gaussian white noise.

A Gaussian white noise with a signal-to-noise ratio of 5 dB is introduced to the current signal to demonstrate the impact of noise on the vibration signal. A comparison is depicted in Figure 15, revealing differences in the amplitude and fluctuation trends of the signals.

**Figure 16:**
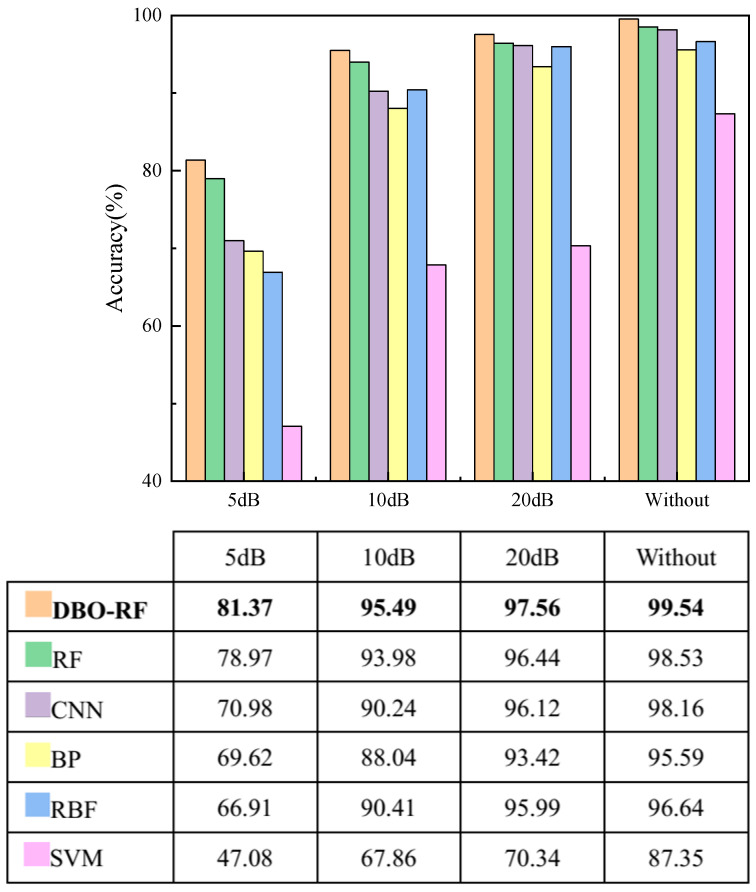
Comparison of the overall test accuracy with different levels of noise.

Gaussian white noise at signal-to-noise ratios of 5 dB, 10 dB, and 20 dB was introduced to the original test dataset to evaluate the overall test accuracy. All methods were assessed at these three noise levels. As illustrated in Figure 16, the performance of all algorithms deteriorates as the noise level increases. Notably, across different noise levels, the proposed algorithm outperforms all other techniques. These results demonstrate that the algorithms presented in this paper exhibit high noise immunity and achieve a consistently high overall test accuracy.

## 7. Conclusions

To tackle the challenges associated with the reliability and stability of fault diagnosis methods in industrial manufacturing PMSMs, a fault diagnosis method based on multi-sensor fusion of image features is proposed. For different types of motor faults, vibration acceleration signals of the PMSM under varying speed and load conditions were collected by sensors placed at different positions. The Gramian matrix is solved separately and the red, green and blue channels are injected to synthesise the final MSDP image. Based on the extraction of image feature vectors through the fusion of Tamura-HOG-LBP texture features, several machine learning methods are compared for the tasks of fault feature learning and classification. The results show that the proposed diagnostic method has the best diagnostic accuracy and robustness, with an average diagnostic accuracy of 99.54%. This technique maximizes the utilization of data collected in industrial settings and enhances its robustness against various environmental conditions by amalgamating multiple sensor signals into an input image. It exhibits outstanding performance, even in noisy environments. The method is non-intrusive and can be extended to condition monitoring and diagnosis of industrial motors, offering prospects for practical industrial applications in motor fault diagnosis. In future work, improvements will be made in the following two areas for better industrial applications. (1) Since PMSMs can encounter load or speed variations during operation, while this paper primarily examines scenarios with constant load and speed, the accuracy of the proposed method may be impacted when applied to situations with load or speed fluctuations. Future research will delve into fault diagnosis for PMSMs in transient load or speed change scenarios. (2) In this study, vibration signals are employed for fusion in fault diagnosis. In the future, the fusion of current, vibration, and temperature signals will be explored to achieve enhanced fault diagnosis capabilities.

## Figures and Tables

**Table 1:** Rated parameters of tested PMSM.

Poles	Voltage	Current	Torque	Speed	Power
4	380 V	1.35 A	2.39 Nm	3000 rpm	750 w

**Table 2:** Parameters of vibration sensor.

Type	Voltage Sensitivity	Frequency Range	Range
A2104E	100 Mv/g	0.5~10 kHz	±50 g pk

**Table 3:** Sample description of the dataset.

Motor Status	Size of the Dataset (Training Set/Validation Set/Testing Set)								
	Speed = 1000 rpm			Speed = 1500 rpm			Speed = 2000 rpm		
	No Load	Half Load	Rated Load	No Load	Half Load	Rated Load	No Load	Half Load	Rated Load
HC	60/20/20	60/20/20	60/20/20	60/20/20	60/20/20	60/20/20	60/20/20	60/20/20	60/20/20
ITSF	60/20/20	60/20/20	60/20/20	60/20/20	60/20/20	60/20/20	60/20/20	60/20/20	60/20/20
LDF	60/20/20	60/20/20	60/20/20	60/20/20	60/20/20	60/20/20	60/20/20	60/20/20	60/20/20
EF	60/20/20	60/20/20	60/20/20	60/20/20	60/20/20	60/20/20	60/20/20	60/20/20	60/20/20
Total	2160/720/720								

**Table 4:** Comparison with other classification methods.

Methods	Accuracy	Precision	Recall	Fl-Score
MGADF+SVM	87.35 ± 2.04	88.76 ± 2.08	87.35 ± 2.04	88.27 ± 2.63
MGADF+BP	95.59 ± 1.35	95.35 ± 1.43	95.59 ± 1.35	95.35 ± 1.54
MGADF+RBF	96.64 ± 1.57	95.36 ± 1.58	96.64 ± 1.57	95.37 ± 1.58
MGADF+CNN	98.16 ± 0.28	98.07 ± 0.25	98.16 ± 0.28	98.07 ± 0.25
MGADF+RF	98.53 ± 0.17	98.45 ± 0.19	98.53 ± 0.17	98.50 ± 0.18
**MGADF+DBO-RF**	**99.54 ± 0.19**	**99.56 ± 0.19**	**99.54 ± 0.19**	**99.58 ± 0.19**

**Table 5:** Diagnostic results of different signal-to-image methods.

Signal-to-Image Method	Accuracy	Precision	Recall	Fl-Score
RP-a	92.28 ± 1.22	92.40 ± 1.22	92.28 ± 1.22	92.28 ± 1.22
RP-b	91.66 ± 0.91	91.71 ± 0.90	91.66 ± 0.91	91.69 ± 0.91
RP-c	92.7 ± 1.40	92.27 ± 1.45	92.7 ± 1.40	92.99 ± 1.42
MTF-a	95.67 ± 1.06	95.77 ± 1.10	95.67 ± 1.06	95.74 ± 1.05
MTF-b	95.43 ± 0.50	95.30 ± 0.52	95.43 ± 0.50	95.27 ± 0.50
MTF-c	95.7 ± 0.78	95.86 ± 0.73	95.7 ± 0.78	95.78 ± 0.76
GASF-a	92.69 ± 0.99	92.96 ± 0.88	92.69 ± 0.99	92.94 ± 0.93
GASF-b	92.47 ± 1.29	92.53 ± 1.26	92.47 ± 1.29	92.50 ± 1.28
GASF-c	92.18 ± 0.52	92.26 ± 0.54	92.18 ± 0.52	92.22 ± 0.53
GADF-a	98.86 ± 0.43	98.88 ± 0.40	98.86 ± 0.43	98.87 ± 0.42
GADF-b	97.33 ± 0.52	97.33 ± 0.62	97.33 ± 0.52	97.33 ± 0.62
GADF-c	97.47 ± 0.21	97.47 ± 0.21	97.47 ± 0.21	97.47 ± 0.21
**MGADF**	**99.54 ± 0.19**	**99.56 ± 0.19**	**99.54 ± 0.19**	**99.58 ± 0.19**

**Table 6:** The average generation time for each image.

Methods	Generation Time per Image
RP	0.1462 s
MTF	0.1832 s
GASF	0.1153 s
**GADF**	**0.1152 s**
MGADF	0.1221 s

## Data Availability

Not applicable.
